# An Efficient Saline-Linked Cautery (SLiC) Method for Robotic Liver Parenchymal Transection Using Simultaneous Activation of Saline-Linked Cautery and Robotic Suctioning: Detailed Technical Aspects and Short-Term Outcomes

**DOI:** 10.7759/cureus.57219

**Published:** 2024-03-29

**Authors:** Takahisa Fujikawa, Yusuke Uemoto, Kei Harada, Taisuke Matsuoka

**Affiliations:** 1 Surgery, Kokura Memorial Hospital, Kitakyushu, JPN

**Keywords:** postoperative complication, robotic anatomical hepatectomy, robotic non-anatomical hepatectomy, saline-linked cautery method, robotic liver resection

## Abstract

Introduction

While there are several advantages to utilizing robotics in liver surgery compared to traditional open and laparoscopic approaches, the most challenging part of robotic liver resection (RLR) remains the liver parenchymal transection. This is primarily due to the constraints of the existing robotic tools and the absence of a standard procedure. This study presents detailed technical aspects of our novel saline-linked cautery (SLiC) method for RLR and assesses the short-term outcomes for both non-anatomical and anatomical RLRs.

Methods

In this study, 82 cases that underwent RLR utilizing the SLiC method at our hospital from September 2021 to December 2023 were examined. A novel SLiC method is introduced in this study for robotically transecting the liver parenchyma utilizing bipolar cautery or monopolar scissors. The technique involves activating the SLiC and robotic suctioning simultaneously. The included patients were divided into two groups: patients undergoing robotic anatomical hepatectomy (n=39), and those receiving robotic non-anatomical hepatectomy (n=43). Short-term outcomes, including intraoperative and postoperative complications, were assessed in patients receiving both anatomical and non-anatomical hepatectomies.

Results

In the whole cohort, 74% of patients had performance status 1 or 2, and 24% were classified as Child-Pugh class B. RLR was performed without Pringle’s maneuver in more than 80% of cases in patients receiving robotic non-anatomical hepatectomy, and more than 80% of patients undergoing robotic anatomical hepatectomy required only four or fewer 15-minute Pringle’s maneuvers. There was no conversion to open hepatectomy, no cases of grade B or C post-hepatectomy liver failure, and no mortality in the entire cohort. Four postoperative complications with CDC IIIa or higher occurred (small bowel obstruction in two cases, intraabdominal hemorrhage in one, and bile leak in another), but no differences in the frequency of complications were found between those undergoing non-anatomical and anatomical hepatectomy (p=0.342).

Conclusions

The SLiC method, which involves simultaneously activating SLiC and robotic suctioning with either monopolar scissors or bipolar cautery, appears to be a secure and convenient technique for liver parenchymal transection in RLR. This innovative method permits precise access to the major Glissonean and venous structures within the liver, making RLR more standardized and easily applicable in routine patient care.

## Introduction

Robotic liver resections (RLRs) have become widely accepted and are now being used in a greater variety of situations. These procedures can be performed with little blood loss, faster recovery times, and reduced postoperative pain compared to traditional open surgeries [1-3]. When comparing traditional laparoscopy to RLRs, the latter offers several advantages, including instruments with seven degrees of freedom, tremor filtration, stable vision in three dimensions, and operation of three instruments from the console [2,4,5]. However, performing liver parenchymal transection during RLR can be challenging because of a shortage of equipment.

An article recently highlighted the innovative saline-linked cautery (SLiC) method in RLR [6-8]. This technique employs superficial thermal coagulation using either monopolar cautery scissors (SLiC-Scissors) or bipolar cautery forceps (Bipolar-SLiC), together with saline supplied from the assistance side. In both procedures, maintaining an adequately moistened condition of the liver cut surface is crucial for continuous dissection and hemostasis.

This study provides detailed technical aspects of the SLiC method for RLR employing simultaneous activation of SLiC and robotic suctioning and assesses the short-term outcomes for both non-anatomical and anatomical RLRs in our institution.

## Materials and methods

This study included 82 RLRs, which were performed at our institution from September 2021 to December 2023. Of those, robotic anatomical hepatectomy (defined as a liver resection carried out along the demarcation line following the occlusion of the Glissonean pedicle) was performed in 39 patients and non-anatomical resections in 43 patients. Patients who had liver tumors greater than 10 cm or tumors being investigated for multi-visceral resection or vascular reconstruction were excluded from the RLR operation. Apart from these criteria, all hepatectomy procedures were planned to be carried out as RLRs.

We collected information on demographics, diagnoses, surgical procedures, and postoperative results by carefully reviewing the surgery database as well as clinic and hospital records. We collected the information prospectively and examined it retrospectively. To evaluate the difficulty level of RLR, we utilized the IWATE criteria [9], in which the difficulty level is ranked on a scale of 0-12. We defined and evaluated postoperative complications using the Clavien-Dindo classification (CDC) [10]. Complications classified as CDC class IIIa or greater were considered to be significant. Operative mortality refers to any deaths occurring within 30 days following surgery.

We assessed the short-term results, safety, and practicality of our technique for robotic liver parenchymal transection. The protocol of the present study (#21021002) was authorized by the Kokura Memorial Hospital Clinical Research Ethics Committee in compliance with the Declaration of Helsinki.

Statistical analysis

Categorical statistics were reported as absolute numbers and percentages, whereas continuous data were represented as the median and range. We utilized Fisher’s exact probability test to compare categorical variables. The statistical significance level was set at a two-sided P-value of 0.05. We performed all statistical analyses using Easy R (EZR; Saitama Medical Center, Saitama, Japan) [11], a user-friendly graphical interface for R (version 2.13.0, R Foundation for Statistical Computing, Vienna, Austria).

Surgical technique

**Figure 1:**
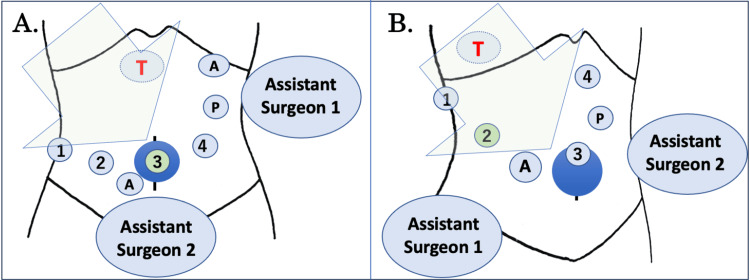
Patient position and trocar placement for robotic liver resection. (A) In the case of the anterolateral (S2, S3, S4b, S5, S6) or superior segments (S8, S4a), the patient was positioned supine with their legs spread apart in a 10° reverse Trendelenburg position, and the four robotic ports were placed at the umbilical level (Figure 1A). In the umbilicus, port #3 was placed through Lap Protector™ with EZ Access™. The ports for the assistant were placed either on the left-upper side or between the #2 and #3 ports (or both). (B) Conversely, in the case of the posterior segment, the patient was positioned in a left lateral decubitus position with the head slightly elevated by 8–10 degrees (Figure 1B). The patient's right side of the abdomen was expanded and stabilized using a flexed operating table. We usually employ five trocars for the procedure. The robotic port #1 was positioned on the right upper lateral side of the abdomen, #3 above the umbilicus, a 12-mm assistance port and #2 port between #1 and #3, and #4 on the left-sided epigastrium. T: the targeted lobe of the liver; A: 12-mm or 5-mm trocars for the assistant surgeon; 1,2,3,4: the four robotic trocars; P: the site of tourniquet for Pringle maneuver; dark blue circle: Lap Protector™ with EZ Access™; big colored arrow: role-in direction of the patient cart. The figure is the authors' own creation.

The Da Vinci Xi surgical system (Intuitive Surgical, Inc., Sunnyvale, CA) was used for every RLR in our series. Figure 1 depicts the patient position and port placement during RLR. In the case of the anterolateral (S2, S3, S4b, S5, S6) or superior segments (S8, S4a), the patient was positioned supine with their legs spread apart in a 10° reverse Trendelenburg position, and the four robotic ports were placed at the umbilical level (Figure 1A). In the umbilicus, port #3 was placed through Lap Protector™ with EZ Access™ (FF0707 or FF1010; Hakko Medical,Tokyo, Japan). The ports for the assistant were placed either on the left-upper side or between the #2 and #3 ports (or both).

Conversely, during the posterior section, the patient was positioned in a left lateral decubitus position with the head slightly elevated by 8-10 degrees (Figure 1B). The patient's right side of the abdomen was expanded and stabilized using a flexed operating table. We usually employed five trocars for the procedure. The robotic port #1 was positioned on the right upper lateral side of the abdomen, #3 above the umbilicus, a 12-mm assistance port and a #2 port between #1 and #3, and #4 on the left-sided epigastrium.

Intraabdominal pressure was maintained at 8 mmHg. The monopolar cautery scissors were connected to the integrated ERBE VIO dV (ERBE USA, Marietta, GA) in forced-coag mode (effect 1, power limit: 40-50 W). The ForceTriad™ (Medtronic PLC, Dublin, Ireland) energy platform was used for the Maryland bipolar forceps with a “standard mode” and an output of 40-50 W. The assistant surgeon was ready to use the ball-tipped SLiC with the electrosurgical VIO device (ERBE Elektromedizin GmbH, Tübingen, Germany). The device was connected to a sterile 0.9% saline bottle, and the drip rate was adjusted to 1-2 cc/minute. Prior to transecting the parenchyma, the Pringle maneuver was set up using the extraperitoneal tourniquet system in a standardized manner outlined in the previous study [12] and was utilized as needed.

Simultaneous Activation of SLiC and Robotic Suctioning

**Figure 2:**
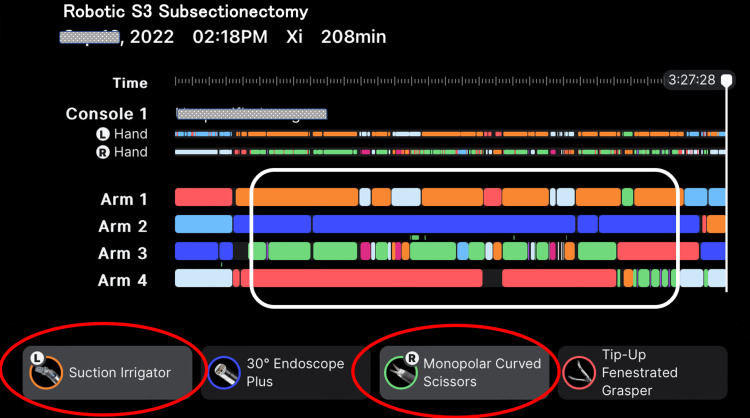
The whole operative process during robotic S3 subsectionectomy. During the SLiC-Scissors method for liver parenchymal transection (the white rounded rectangle), the procedure was mostly performed using a suction irrigator (red oval) in the left hand and monopolar curved scissors (red oval) in the right hand (the figure is created and modified using iPhone application of “My Intuitives” provided by Intuitive Surgical G.K.). S3: segment 3; SLiC: saline-linked cautery

**Figure 3:**
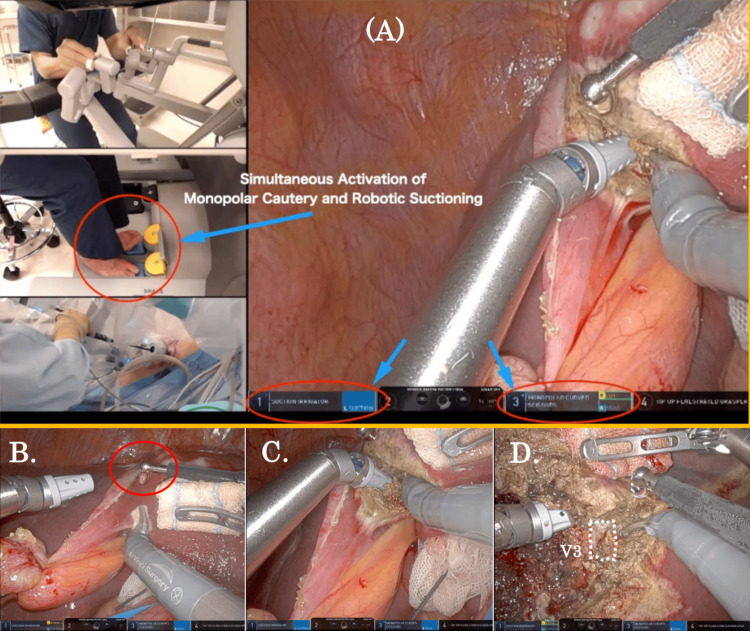
The SLiC method using simultaneous activation of SLiC and robotic suctioning during robotic S3 subsectionectomy. (A) SLiC was activated simultaneously with robotic suctioning by using both legs. (B) Liver parenchymal transection took place under saline-dripping from the assistant's side. (C) The surface layer of the deep fissure could be continuously boiled and coagulated, and rapid hemostasis was achieved. (D) In the case of parenchymal transection around the V3, the short-pitch dissection around the vein using the tip of the scissors was performed. SLiC: saline-linked cautery, S3: segment 3; V3: the venous branch in S3.

Video 1 and Figures 2-3 illustrate the overall framework of simultaneous activation of SLiC and robotic suctioning in the SLiC method. When the SLiC method was used to transect the liver parenchyma, the procedure was mostly performed using a suction irrigator in the left hand and monopolar curved scissors in the right hand (Figure 2). SLiC was activated simultaneously with robotic suctioning by using both legs (Figure 3A). When performing the dissection and controlling bleeding, we used low-temperature thermal coagulation of the superficial dissection surface by dripping saline from the assistance side (Figure 3B). The suction irrigator effectively maintained the operative field, which was expanded and properly hydrated. The multi-joint monopolar curved scissors were used to scrape liver parenchyma back and forward to consistently carry out thin-layer dissection (Figure 3C). Alternatively, the parenchyma was scraped by the short-pitch movement of the monopolar scissors in the case of parenchymal transection around the major vessels (Figure 3D).

**Video 1:** The SLiC method using simultaneous activation of SLiC and robotic suctioning during robotic S3 subsectionectomy. SLiC: saline-linked cautery; S3: segment 3

Liver Parenchymal Transection Using the SLiC-Scissors Method

**Figure 4:**
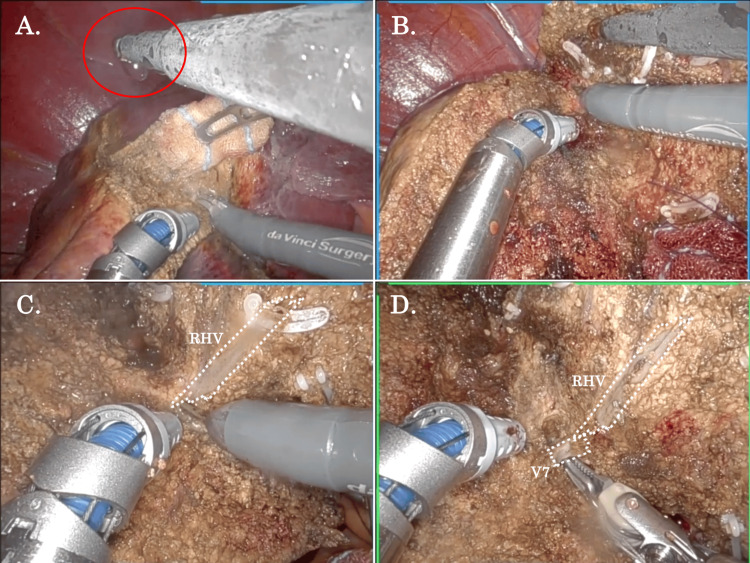
The SLiC-Scissors method using the monopolar curved scissors during robotic right posterior sectionectomy. (A) The saline was dripped from the assistant's side, and the liver parenchyma was scraped back and forward using the tip of the multi-joint monopolar scissors to continuously promote thin-layer dissection. (B) The minor bleeding was controlled by continuous low-temperature coagulation under adequate moisture. (C) In the case of parenchymal dissection around the right hepatic vein (RHV), short-pitch dissection using the tip of the monopolar scissors was applied to avoid the pullout of small venous branches. (D) The parenchymal transection around the RHV was also securely advanced to expose V7. SLiC: saline-linked cautery, RHV: right hepatic vein, V7: the venous branch in S7.

The SLiC-Scissors method using monopolar scissors during robotic right posterior sectionectomy is shown in Figure 4 and Video 2. The saline was dripped from the assistant side, and the liver parenchyma was scraped back and forward using the tip of the multi-joint monopolar scissors to continuously promote thin-layer dissection (Figures 4A, 4B). The minor bleeding could be controlled by continuous low-temperature coagulation under adequate moisture. During the dorsal-side parenchymal dissection via a caudal view, the SLiC method was also easily applied. In the case of parenchymal dissection around the right hepatic vein (RHV), short-pitch dissection using the tip of the monopolar scissors was applied to avoid the pullout of small venous branches (Figure 4C). The multi-joint and tremor-filtration functions of the arms facilitated the meticulous parenchymal dissection. The parenchymal transection around the RHV was also securely advanced to expose the venous branch of Segment 7 (Figure 4D).

**Video 2:** The SLiC-Scissors method using the monopolar cautery scissors during robotic right posterior sectionectomy. SLiC: saline-linked cautery.

Liver Parenchymal Transection Using the Bipolar-SLiC Method

**Figure 5:**
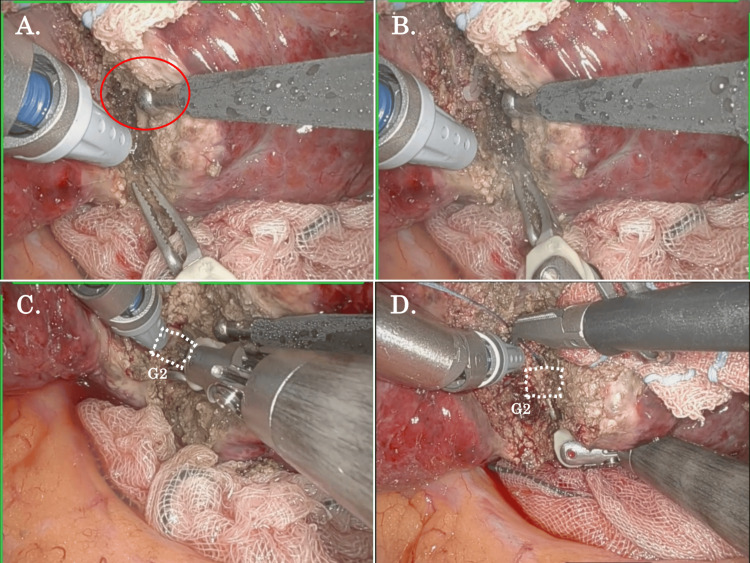
The Bipolar-SLiC method using the Maryland Bipolar Forceps during robotic S2 subsectionectomy. (A) The saline was supplied from the assistant side (red oval), and the Bipolar-SLiC method was used during liver parenchymal transection along the G2 to avoid heat injury to the major Glissonean structures. (B) Short-pitch dissection was applied to expose the G2 and the minor bleeding was controlled by continuous low-temperature coagulation from the bipolar cautery under adequate moisture. (C) Meticulous dissection was performed on the upper, lower, and back of G2. (D) After sufficient parenchymal dissection along the G2, it was encircled, ligated, and clipped without any major bleeding events. SLiC: saline-linked cautery, G2: S2 Glissonean branch.

The Bipolar-SLiC method using the Maryland bipolar forceps during robotic S2 subsectionectomy is shown in Figure 5 and Video 3. To avoid heat injury to the major Glissonean structures, the bipolar-SLiC method was used during liver parenchymal transection along the S2 Glissonean branch (G2). All the procedures regarding parenchymal transection could be performed without using Pringle’s maneuver. The saline was supplied from the ball-tipped saline-linked monopolar cautery, which was manipulated by the assistant surgeon (Figure 5A). Short-pitch dissection was applied to expose the G2, and the minor bleeding could be controlled by continuous low-temperature coagulation from the bipolar cautery under adequate moisture (Figure 5B). Using the bipolar-SLiC method, meticulous dissection could be performed on the upper, lower, and back of G2 (Figure 5C). After sufficient parenchymal dissection along the G2, it was encircled, ligated, and clipped without any major bleeding events (Figure 5D).

**Video 3:** The Bipolar-SLiC method using the Maryland Bipolar Forceps during robotic S2 subsectionectomy. SLiC: saline-linked cautery.

## Results

In the whole cohort, the median age was 74 years (range 44 to 88 years), 74% of patients were performance status 1 or 2, and 24% were classified as Child-Pugh class B. Of the 82 cases, 44 (54%), 31 (38%), and 5 (6.1%) were hepatocellular carcinoma, colorectal cancer liver metastasis, and intrahepatic cholangiocarcinoma, respectively. There was no conversion to open hepatectomy, no cases of grade B or C post-hepatectomy liver failure, and no mortality in the entire cohort. Four postoperative complications with CDC IIIa or higher occurred, but no differences in the frequency of complications were found between those undergoing non-anatomical and anatomical hepatectomy (p=0.342). In addition, we experienced no cases of bile duct stenosis during the follow-up period of 3-30 months.

Table 1 displays the surgical factors and outcomes in patients undergoing robotic non-anatomical hepatectomy (n=43). Median operative and console time was 227 and 133 min, respectively. Only one patient (2.3%) experienced surgical blood loss of 200 mL or more, and no patient required an intraoperative red blood cell (RBC) transfusion. RLR was performed without Pringle’s maneuver in more than 80% of cases. There was one patient with a CDC IIIb postoperative complication. The patient receiving repeat liver resection and extensive adhesiolysis suffered from intraabdominal hemorrhage and required re-operation. The bleeding occurred at the site of adhesiolysis, not from the liver resection surface.

**Table 1:** Surgical factors and outcomes in patients undergoing robotic non-anatomical hepatectomy in the current cohort (n=43). ^*^P-values < 0.05 were considered significant. RBC, red blood cell; P-maneuver, Pringle’s maneuver; CDC, Clavien-Dindo Classification; PHLF, post-hepatectomy liver failure; LOS, length of postoperative stay.

Variables	Values
Difficulty score; median (range)	5 (1-7)
Operative time; min, median (range)	227 (90-613)
Console time; min, median (range)	133 (30-442)
Blood loss; mL, median (range)	5 (5-400)
Blood loss of 200 mL or more; n (%)	1 (2.3)
RBC transfusion; n (%)	0 (0.0)
Duration for P-maneuver; min, median (range)	0 (0-165)
No P-maneuver performed; n (%)	35 (81.4)
5 or more times for 15-min P-maneuver; n (%)	1 (2.3)
Conversion to open surgery; n (%)	0 (0.0)
Mortality; n (%)	0 (0.0)
Complications (CDC IIIa or higher); n (%)	1 (2.3)
Intraabdominal bleeding	1 (2.3)
PHLF (Grade B, C)	0 (0.0)
LOS; days, median (range)	7 (4-27)

Table 2 shows the surgical factors and outcomes in patients undergoing robotic anatomical hepatectomy (n=39). Median operative and console time were 448 and 344 min, respectively. Two patients (5.1%) experienced surgical blood loss of 200 mL or more, but no patients required an intraoperative RBC transfusion. In this group, 82% of patients required only four or fewer 15-minute Pringle’s maneuvers. Three postoperative complications with CDC IIIa or higher occurred (small bowel obstruction in two cases and bile leakage in one). Concerning small bowel obstruction, one patient underwent simultaneous resection of ascending colon cancer and an S5 metastatic liver tumor, and the other had previously undergone laparotomy and extensive adhesiolysis. Postoperative bile leakage was observed in a patient who underwent robotic S8 liver resection, but it improved conservatively.

**Table 2:** Surgical factors and outcomes in patients undergoing robotic anatomical hepatectomy in the current cohort (n=39). *P-values < 0.05 were considered significant. RBC, red blood cell; P-maneuver, Pringle’s maneuver; CDC, Clavien-Dindo Classification; PHLF, post-hepatectomy liver failure; LOS, length of postoperative stay.

Variables	Values
Difficulty score; median (range)	7 (4-11)
Operative time; min, median (range)	448 (199-768)
Console time; min, median (range)	344 (143-616)
Blood loss; mL, median (range)	24 (5-400)
Blood loss 200 mL or more; n (%)	2 (5.1)
RBC transfusion; n (%)	0 (0.0)
Duration for P-maneuver; min, median (range)	30 (0-165)
No P-maneuver performed; n (%)	12 (30.8)
5 or more times for 15-min P-maneuver; n (%)	7 (17.9)
Conversion to open surgery; n (%)	0 (0.0)
Mortality, n (%)	0 (0.0)
Complications (CDC IIIa or higher); n (%)	3 (7.7)
Small bowel obstruction	2 (5.1)
Bile leak	1 (2.6)
PHLF (Grade B, C)	0 (0.0)
LOS; days, median (range)	8 (5-48)

## Discussion

The current study describes an effective SLiC method for transecting liver parenchyma during RLR by simultaneously activating SLiC and robotic suctioning. This method allows for quick and safe liver parenchymal transection with minimal need for applying Pringle’s maneuver during RLR. Moreover, it can be achieved without raising the danger of bile leakage or bile duct stenosis during the follow-up period of 3-30 months, even during robotic anatomical liver resection.

Based on the available information, the primary drawback of RLR is the transection of the liver parenchyma. We recently reported the implementation of a novel RLR method utilizing the SLiC [6-8]. The SLiC approach in RLR uses superficial thermal coagulation using either monopolar scissors or bipolar cautery, together with saline dropping from the assistant's side [6]. The present approach follows the "Kyoto University-style liver parenchymal transection" technique used in open hepatectomy. This involves low-temperature thermal coagulation (100 degrees Celsius or below) on the surface layer by saline-linked bipolar cautery [13,14]. The current procedure enables the cautery tip to stay clean by employing saline, allowing for quick and precise dissection and achieving hemostasis at the same time. In addition, the SLiC method can utilize either monopolar curved scissors (SLiC-Scissors) or bipolar cautery forceps (Bipolar-SLiC) when the cautery is activated simultaneously with robotic suctioning, and the method is safe and practicable during both non-anatomical and anatomical RLR.

We initially introduced the SLiC-Scissors method as a novel robotic liver parenchymal transection technique using saline-linked monopolar scissors [9]. With robotic systems that include tremor-filtration and motion-scaling abilities, we can confidently manipulate sharp-edged scissors for precise, thin-layered liver parenchymal transection while ensuring safety. Moreover, we regularly utilize the saline-linked cautery combined with the wet oxidized cellulose (SLiC-WOC) technique during laparoscopic liver resection [15,16]. This treatment successfully handles most instances of intractable bleeding from deep liver parenchymal fissures by transporting superficial boiling heat coagulation via contact with sodium chloride solution to the bleeding point by inserting wet oxidized cellulose. Using saline-linked monopolar scissors, the SLiC-WOC method can also be applied to the robotic liver parenchymal transection, and rapid hemostasis for intractable bleeding can be achieved.

In contrast, the Bipolar-SLiC method, which uses saline instillation in combination with the clump-crushing method, is somewhat difficult to deal with bleeding from the deep parenchymal fissure, and consequently, the speed of liver parenchymal transection tends to be slower than the SLiC-Scissors method. Due to the bipolar cautery’s nature, it is also disadvantageous not to utilize the SLiC-WOC method for intractable bleeding. On the other hand, it has the advantage of being relatively easy to operate due to the shape of the tip, making it easy for beginners to handle. Additionally, compared to the monopolar cautery scissors, the bipolar cautery forceps have a more limited spread of thermal coagulation, making it less likely that thermal damage to deep tissues will occur. Therefore, it can be safely used during parenchymal dissection around the major Glissonean structures during anatomical resection. In particular, as shown in this study, by performing short-pitch hepatic parenchymal dissection with careful bleeding control, it is relatively easy to secure large blood vessels intrahepatically.

One thing to be aware of when using the Bipolar-SLiC method is that the wire in the joint may be heat-damaged through the moisture dripping during intraoperative activation. The joints of the monopolar curved scissors are protected by an insulator, but in Maryland bipolar forceps, there is no insulator, and the wires are exposed. For this reason, if there is high power to be supplied to the bipolar cautery and too much moisture around the joint, a current short-circuit will be formed at the naked wire level when electricity is applied, resulting in critical heat damage to the wire. If it occurs, the majority of the cases are not clinically relevant, but the bipolar forceps will be broken and will not be used. To avoid this, the power supplied to the bipolar cautery is set lower (40-50 W) than that in the double bipolar method [17], and the water supplied to the surgical field must be localized to the liver transection surface and the bipolar tip as much as possible.

Some limitations exist in the current investigation. Firstly, since the study was performed retrospectively, its usefulness in assessing how treatments impact outcomes is somewhat limited. Second, the number of the sample was relatively small, and increasing the sample size will be helpful in producing more reliable recommendations. Finally, to truly show that our suggested approach is better than other options, we need to include a control group. We firmly believe that this strategy is a positive step towards standardizing RLR in the future. This could result in enhanced security and better outcomes, marking another stride towards the extensive use of RLR.

## Conclusions

We report an efficient technique for liver parenchymal transection in RLR using the SLiC method. Using simultaneous activation of SLiC and robotic suctioning, rapid and secured liver parenchymal transection can be achieved with minimal application of Pringle’s maneuver during RLR. Moreover, it can be achieved without raising the chances of bile leakage or bile duct stenosis. This method can provide a safe and efficient way to do liver parenchymal transection in RLR.
